# Neural Stem Cell-Based Therapies and Glioblastoma Management: Current Evidence and Clinical Challenges

**DOI:** 10.3390/ijms22052258

**Published:** 2021-02-24

**Authors:** Amira Yasmine Benmelouka, Malak Munir, Ahmed Sayed, Mohamed Salah Attia, Mohamad M. Ali, Ahmed Negida, Badrah S. Alghamdi, Mohammad Amjad Kamal, George E. Barreto, Ghulam Md Ashraf, Mostafa Meshref, Eshak I. Bahbah

**Affiliations:** 1 Faculty of Medicine, University of Algiers, Algiers 16000, Algeria; amira.yasmine.benmelouka@gmail.com; 2 Faculty of Medicine, Ain Shams University, Cairo 11591, Egypt; malakmounir@gmail.com (M.M.); ahmedsayed8991@gmail.com (A.S.); 3 Department of Pharmaceutics, Faculty of Pharmacy, Zagazig University, Zagazig 44519, Egypt; mosalahnabet@gmail.com; 4 Faculty of Medicine, Al-Azhar University, Damietta 34511, Egypt; Mohamed.Ali@domazhermedicine.edu.eg (M.M.A.); isaacbahbah@gmail.com (E.I.B.); 5 School of Pharmacy and Biomedical Sciences, University of Portsmouth, Portsmouth PO1 2UP, UK; ahmed.said.negida@gmail.com; 6 Faculty of Medicine, Zagazig University, Zagazig 44519, Egypt; 7 Department of Physiology, Neuroscience Unit, Faculty of Medicine, King Abdulaziz University, Jeddah 21589, Saudi Arabia; basalghamdi@kau.edu.sa; 8 Pre-Clinical Research Unit, King Fahd Medical Research Center, King Abdulaziz University, Jeddah 21589, Saudi Arabia; ashraf.gm@gmail.com or; 9 West China School of Nursing/Institutes for Systems Genetics, Frontiers Science Center for Disease-Related Molecular Network, West China Hospital, Sichuan University, Chengdu 610041, China; prof.ma.kamal@gmail.com; 10 King Fahd Medical Research Center, King Abdulaziz University, P. O. Box 80216, Jeddah 21589, Saudi Arabia; 11 Novel Global Community Educational Foundation, 7 Peterlee Place, Hebersham, NSW 2770, Australia; 12 Department of Biological Sciences, University of Limerick, V94 T9PX Limerick, Ireland; 13 Instituto de Ciencias Biomédicas, Universidad Autónoma de Chile, Santiago 32310, Chile; 14 Department of Medical Laboratory Technology, Faculty of Applied Medical Sciences, King Abdulaziz University, Jeddah 21589, Saudi Arabia; 15 MSc Neurology, Al-Azhar University, Cairo 11651, Egypt; mostafameshref1988@gmail.com

**Keywords:** stem cells, glioblastoma, neuro-oncology, recombinant vectors, neural stem cells, cancer

## Abstract

Gliomas, which account for nearly a quarter of all primary CNS tumors, present significant contemporary therapeutic challenges, particularly the highest-grade variant (glioblastoma multiforme), which has an especially poor prognosis. These difficulties are due to the tumor’s aggressiveness and the adverse effects of radio/chemotherapy on the brain. Stem cell therapy is an exciting area of research being explored for several medical issues. Neural stem cells, normally present in the subventricular zone and the hippocampus, preferentially migrate to tumor masses. Thus, they have two main advantages: They can minimize the side effects associated with systemic radio/chemotherapy while simultaneously maximizing drug delivery to the tumor site. Another feature of stem cell therapy is the variety of treatment approaches it allows. Stem cells can be genetically engineered into expressing a wide variety of immunomodulatory substances that can inhibit tumor growth. They can also be used as delivery vehicles for oncolytic viral vectors, which can then be used to combat the tumorous mass. An alternative approach would be to combine stem cells with prodrugs, which can subsequently convert them into the active form upon migration to the tumor mass. As with any therapeutic modality still in its infancy, much of the research regarding their use is primarily based upon knowledge gained from animal studies, and a number of ongoing clinical trials are currently investigating their effectiveness in humans. The aim of this review is to highlight the current state of stem cell therapy in the treatment of gliomas, exploring the different mechanistic approaches, clinical applicability, and the existing limitations.

## 1. Introduction

Gliomas are a group of primary tumors of the central nervous system (CNS) originating from glial cells [1]. According to the Central Brain Tumor Registry of the United States (CBTRUS), gliomas account for around 25% of all primary brain tumors, with glioblastoma being the most common glioma, as well as the most common malignant CNS tumor [2]. Histologically, gliomas originate from three types of glial cells: Oligodendrocytes, ependymal cells, and astrocytes. The most common histological variant of glioma is astrocytic tumors accounting for over 70% of all gliomas [2]. The most recent World Health Organization (WHO) classification of gliomas is based on both histological—according to their cell of origin (e.g., astrocytoma, ependymoma)—and molecular—according to specific acquired mutations (e.g., diffuse astrocytoma, IDH-mutant)—characteristics [3]. Tumors are also graded on a scale from one to four, with grade IV glioblastomas being the most invasive and lethal [3]. In contrast to low-grade gliomas, in which concomitant chemotherapy is not always required [4,5], the infiltrative and diffuse nature of high-grade “malignant” gliomas mandates the use of chemotherapy [6].

The management of malignant gliomas poses several challenges, in part due to the heterogeneous and resistant nature of neoplasm, as well as the obstacles faced when administering high-dose radiation and chemotherapy in tissue as vulnerable as that of the CNS. Limitations to therapy also include the unfavorable pharmacokinetics of chemotherapeutic drugs, which prevent them from efficiently penetrating the blood–brain barrier, and frequent relapses due to the metastatic seeding associated with glioblastoma [7,8]. Shortcomings in the current treatment options for malignant gliomas have sparked an interest in the search for novel techniques such as direct receptor antagonists, immune therapy, and stem cell therapy. 

Stem cells (SCs) are precursor cells that retain the capacity to differentiate into various types of tissues. Stem cells are classified according to their origin; however, adult stem cells, such as mesenchymal stem cells (MSCs), are the most commonly used therapeutically. MSCs are multipotent stem cells that can differentiate into all cells of a mesenchymal lineage [9,10] and are isolated from the bone marrow, adipose tissue, umbilical cord, and dental pulp. Neural stem cells (NSC) are specific types of adult stem cells found in the subependymal zone and the dentate gyrus, and are responsible for the regeneration of neurons, astrocytes, and oligodendrocytes [11]. 

Stem cell therapy involves either the administration of exogenous stem cells or the mobilization of endogenous stem cells. Stem cell mobilization is an important approach in the management of degenerative disorders, whereas the administration of exogenous stem cells is more pertinent in the management of malignant gliomas. Several studies have demonstrated the ability of stem cells to target brain pathologies, such as areas of demyelination, ischemia, and neoplasms [7,12,13]. Both MSCs and NSCs were found to have high tropism to malignant gliomas due to the overexpression of cell surface markers, as well as the secretion of molecular signals in the tumor’s microenvironment [14,15]. Factors such as cytokines (e.g., tumor necrosis factor alpha “TNF-α,” interkelukin-8 “IL-8,” and stromal cell-derived factor alpha “SDF-α”) [14,16,17,18], hypoxia-inducible factor-1a, hepatocyte growth factor, and vascular endothelial growth factor have all been implicated in stem cell migration toward neoplasms [19,20], as well as tumor extracellular matrix components such as tenascin-C, laminin, and inhibitor of matrix metalloproteinase-1 [19,21]. 

**Figure 1:**
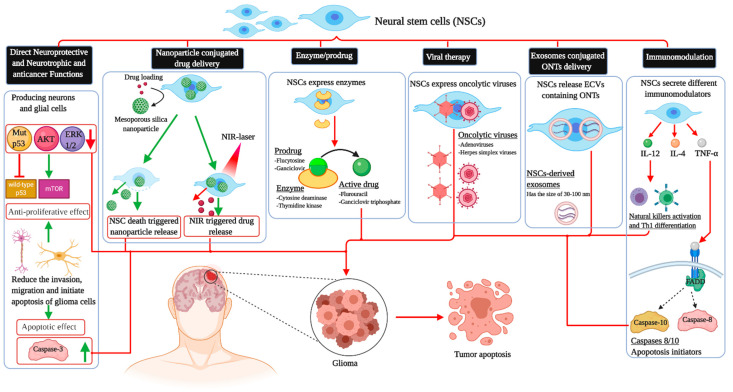
The different applications of neural stem cells in killing glioblastoma cells. NSCs: Neural stem cell; AKT: Protein kinase B; ERK1/2: Extracellular-regulated kinase; TNF-α: Tumor necrosis factor alpha; IL-4: Interkelukin-4; IL-12: Interkelukin-12; FADD: FAD-associated protein with death domain; ONTs: Oligonucleotide therapeutics; Th1: T helper 1; mTOR: Mammalian target of rapamycin; NIR: Near-infrared; ECVs: Extracellular vesicles.

This intrinsic property of stem cells has prodded interest in their ability to serve as drug delivery systems, which could potentially circumvent the blood–brain barrier. In vivo animal studies have shown that the modification of stem cells can directly target neoplasms through various mechanisms, decrease the tumor burden, and thus prolong survival. Molecularly engineered stem cells can be modified to (1) prevent angiogenesis, (2) deliver inflammatory cytokines and mediate immune response, (3) initiate ligand-activated anti-tumor pathways, (4) compete for certain pro-proliferative ligands and thus inhibit tumor growth, (5) release anti-tumor toxins, (6) induce “cell suicide” through well-established enzyme-prodrug systems, (7) deliver nanoparticles and oncogenic viral particles, and finally, (8) release vesicles containing anti-tumor microRNA (miRNA) (Figure 1) [7].

In this review, we aim to provide an overview of the current state of stem cell therapy in the treatment of gliomas. The articles chosen for inclusion in this review were those investigating the effects of stem cell therapy in both human and animal trials, as well as additional articles outlining the mechanisms of action of the various trialed modalities through experimentation. We will explore the intrinsic properties of stem cells that make an attractive treatment option, as well as the various mechanisms that have been experimentally explored. In addition, we aim to provide a brief synopsis of the applicability of this approach in humans, as well as the limitations thereof. 

## 2. Tropism, Migration, and Tumor Homing Properties of Neural Stem Cells

NSCs are mainly detected in the hippocampus and the subventricular zone situated in the dentate gyrus of the brain [22,23]. Thanks to their tropic properties, they can serve as delivery vehicles of a variety of elements such as antitumor drugs and suicide genes in a selective way to the tumoral mass [24]. Therefore, NSCs have been extensively investigated in drug and oncolytic viruses delivery in brain malignancies, especially medulloblastomas [25] and gliomas [26,27,28]. This tropism was explored in rodent brains through the simultaneous NSCs and glioblastoma cells implementation [29]. NSCs have the ability to migrate toward malignant brain masses of glial origin and tumors of other origins such as medulloblastoma and metastatic cancers such as melanoma and breast neoplasms [25,30,31]. 

The migratory movement of NSCs starts about 50 min after their transplantation, and the number of stem cells in the tumor site increases slowly up to 5 days in the region, with a significant expansion up to 15 days later [32]. This migration of NCSs to the malignant mass may progress in a dose-dependent manner [33], and it is under the influence of the tumoral microenvironment components [34]. Furthermore, the killing capacity may also be influenced by the distance between the delivery site and the tumor. A recent report showed that direct injection of the potent stem cells into the tumor foci led to a rapid decrease in tumor growth with a reduction in the mass volume to sub-detection levels after ten days post-NCSs delivery, whereas implementation at a distance of two millimeters far from the mass was associated with a significant attenuation of tumor proliferation by day 14 and a reduction in the mass to sub-detection level by day 21 after NSC delivery [34]. 

**Figure 2:**
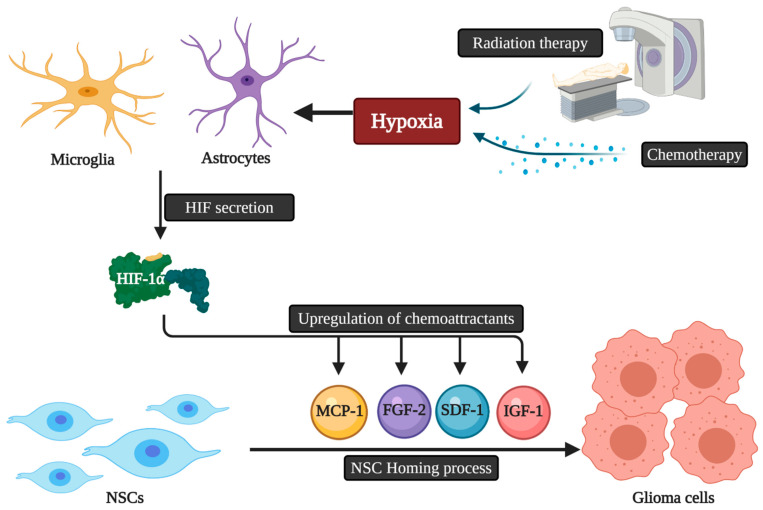
The possible relation of chemoradiotherapy and HIF-1α secretion with NSCs homing to glioblastoma. NSCs: Neural stem cells; HIF: Hypoxia-inducible factor; IGF1: Insulin-like growth factor 1; SDF-1: Stromal cell-derived factor 1; VEGF: Vascular endothelial growth factor; MCP1: Monocyte chemotactic protein 1; FGF2: Fibroblast growth factor 2.

The migratory capacity of NCSs is dependent on chemotactic factors. Accordingly, the presence of multifocal masses may reduce the killing capacities of NSC therapies due to the decrease in the amount of NSCs reaching each mass, as each focus releases chemotactic factors and thus may dilute the NSC dose per tumor mass [34]. The exact mechanistic pathways that guide NSCs homing to gliomas are still unknown. Microglia and astrocytes secrete a variety of angiogenic and inflammatory agents that play a role in NSC homing [35]. These homing properties can also be triggered by hypoxia via the secretion of a key element called the Transcription Factor Hypoxia-Inducible Factor-1α (HIF-1α). HIF-1α can promote the upregulation of chemoattractant substances, including various chemokines and other molecules acting as growth factors like insulin-like growth factor 1 (IGF1), stromal cell-derived factor 1 (SDF-1), vascular endothelial growth factor (VEGF), monocyte chemotactic protein 1 (MCP1), and fibroblast growth factor 2 (FGF2) [36]. The down-regulation of HIF-1α in glioblastoma cells leads to the decline of SDF-1 and the expression of VEGF with NSC tumor tropism suppression [37]. Finally, as conventional cancer therapies, namely radiation and chemotherapy, are associated with the hypoxia-induced upregulation of chemokines by malignant cells, the use of these therapies concomitantly with NSC delivery may enhance the chemotactic pathways and signals that potentiate stem cell migration and thus allow improved overall therapeutic efficacy [38] (Figure 2).

## 3. Neuroprotective and Neurotrophic Functions of Neural Stem Cells Therapy

Endogenous subventricular stem cells have the ability to divide and to migrate to the injured site in stroke and other CNS injuries. They can also undergo a process of differentiation resulting in mature cells that can participate in the recovery [39,40,41,42]. The application of NSCs is a promising research avenue due to their potential in improving the outcome of CNS injury and neurodegeneration [43]. The multipotency and self-regenerative attributes of NSCs are crucial for nervous tissue repair [44]. NSCs are thus considered as an ideal source to continuously produce glial cells and neurons to repair neural networks in the damaged nervous system [45]. NSCs can enhance the recovery from brain injury through their migration and cell replacement properties, in addition to the enhancement of nutritional and trophic supplementation effects using paracrine processes [46,47]. They can also control inflammation in the brain and provide some neuroprotective activities [48,49,50]. Moreover, NSC therapies can also positively influence intracranial blood perfusion via promoting angiogenesis as they can increase angiogenic factors expression in the brain [51,52]. In addition to their numerous advantages, in vivo studies have shown that intravenously administered NSCs can cross the blood–brain barrier [53,54] and exert their activities without producing toxicity in the normal components of the brain [55]. Furthermore, the ability of NSCs to cross the blood–brain barrier has been closely linked to the expression of certain cell surface adhesion molecules such as CD44, VLA-4 [56], as well as the inflammatory state of the CNS. In an in vivo study by Pluchio et al., tagged NSCs injected intravenously were detected in the CNS in mice pre-treated with lipopolysaccharide or tumor necrosis factor and interleukin 1β—inflammatory mediators used to mimic an inflammatory-like state [56]. More specifically, in an in vivo model of gliomas, the expression of VEGF, HGF, and zonulin—factors that increase the permeability of the blood–brain barrier—induced transmigration of the NSCs to the CNS after being injected into the systemic circulation [57]. In addition, data from recent reports showed that the systemic stem cells’ administration efficiency was much higher in animals with neurodegeneration than wild-type animals [56,58]. The in vivo-tracking of NSCs homing to glioblastoma using immuno-histochemical studies revealed that the systemically administrated progenitor cells can cross the barrier and localize in glioblastoma foci [54,59]. The quantitative optical analysis showed that, when the intravenous route is used, about 1.4% of NSCs co-localized with the tumor while the intraventricular delivery resulted in the localization of more than 4% of NSCs [54]. Politi et al. monitored the accumulation of intravenously administered NPCs in a model of autoimmune encephalomyelitis using a human magnetic resonance scanner. They could detect the transplanted cells in about 80% of the brain lesions 24 h after the injection. The continued assessment showed the presence of NPCs 20 days after the injection. The neuropathological study of the brains showed that the transplanted stem cells were exclusively in inflammatory regions of neurodegeneration and not in normal tissue, suggesting their potential role in the reversal of the inflammatory process [60]. Finally, the implementation of exogenous human NSCs into the dentate gyrus can also activate the production of endogenous NSCs [58].

## 4. Effects of Neural Stem Cells in Glioma

The subcutaneous injection of normal NSCs and human glioma (U251) cell lines in nude mice led to the promotion of the animals’ survival [59]. This observation was concomitant with a decline in mutant p53 production and phosphorylation of protein kinase B (AKT) and extracellular-regulated kinase (ERK1/2). A significant increase in an important apoptotic molecule called caspase-3 was also noted, suggesting that normal NSCs may exert direct effects against malignant glioma [59]. In another report, cultures containing U87 stem-like cells in contact with an NSC-conditioned medium showed low viability and multiplication of U87 cells without significant modulation of their astrocytic differentiation capacity. Moreover, the invasive and migratory functions of U87 stem-like cells were also reduced [60]. 

It was also established that endogenous normal stem cells belonging to the subventricular zone can also target glioma-proliferating cells and attenuate the mass growth with a potential impact on survival [61]. Vitamin K-dependent factor protein S, released by the tumoral environment, can trigger this specific tropism via the modulation of the tyrosine kinase receptor (Tyro3) action [62].

## 5. Immunomodulation

As previously mentioned, genetic alteration of an NSC can induce the production of anti-neoplastic compounds near the tumor; one such class is immunomodulators. For instance, investigators have programmed NSCs to express IL-4 and IL-12 in separate experiments [63,64]. Both approaches have succeeded in reducing tumor burden, as well as prolonging survival in mice. IL-12 is a known stimulator of T-cells that not only activates natural killer cells, but also induces differentiation of T-cells to the Th1 subtype of CD4+ T-cells [65]. Accordingly, Ehtesham et al. showed that the improved survival seen upon injecting IL-12-secreting NSCs was associated with a greater degree of tumor infiltration by CD4+ and CD8+ T-cells [64]. IL-4 has been shown to enhance the recruitment of precursor T-cells, thus enhancing the immune response against the tumor [66]. Using IL-4, Benedetti et al. demonstrated the efficacy of IL-4 as a tumor-combating cytokine, and also showed that delivering it specifically via NSCs led to improved survival durations compared to introducing IL-4 using other methods, such as retroviral transfer. While retroviral transfer of IL-4 did prolong mice survival compared to the control group, it was still significantly less beneficial than introducing the same cytokine using NSCs, suggesting that the intrinsic anti-tumor activity of NSCs may, in part, be responsible for the improvements [63]. In addition, other immunomodulators, including different interleukins (IL-23 and 24), as well as interferon-β, have also been used [64,67,68,69,70]. IL-23 has been shown to stimulate the lymphocytic response against gliomas—mainly by inducing CD8^+^-cells—but also partially through inducing CD4^+^ and NK cells. There is some evidence that this response may be mediated through increased IFN-γ expression, as its mRNA was found to be upregulated in the related experiment [69]. IL-24, on the other hand, induces a pro-apoptotic effect by suppressing the translation of anti-apoptotic proteins such as BCL-XL, as well as promoting the transcription of certain pro-apoptotic proteins, such as GADD34 [71]. Interferon-β, through its differential effects on the expression of certain genes, induces an apoptotic and immune response in addition to the inhibition of angiogenesis [72].

Another promising molecule is the TNF-related apoptosis-inducing ligand (TRAIL), which by binding death receptors (DR) 4 and 5, can induce apoptosis in malignant cells [73]. By binding DR4/5, TRAIL induces them to recruit Fas-associated protein with death domain (FADD), which then binds to caspases 8/10 [74,75]. Caspase 8/10 are both well-known initiators of apoptosis (known as “initiator” caspases) that induce cell death [76,77]. Animal studies have documented an inhibition of tumor growth, as well as increased rates of apoptosis, as a result of NSCs that are genetically altered to express TRAIL in combination with other synergistic substances discussed below [78,79,80]. 

The use of a secretory form (denoted S-TRAIL), in combination with complementary anti-tumor substances, can further enhance its ability to combat tumors. For instance, by using short hairpin RNA (shRNA), we can silence the genetic expression of Bcl2, a known anti-apoptotic protein [79]. Alternatively, we can inhibit microRNA 21, which is known to promote Bcl2 expression [81]. Either of the two approaches can further potentiate the pro-apoptotic effect of TRAIL, and thereby increase efficacy.

An alternative method involves hyper-sensitizing the tumor cells to S-TRAIL such that it exerts maximal apoptotic effects. Combining TRAIL with proteasome inhibitors leads to the upregulation of DR5 expression—the target receptor of TRAIL. Proteasome inhibitors generate reactive oxygen species (ROS), which trigger p53 binding to regulatory introns of the DR5 gene and increase its expressions levels [82]. Furthermore, inhibitors of nuclear factor kappa-beta (NF-κB) were decreased, and similar to p53, binding of NF-κB to the intron gene of DR5 upregulates its expression. Increased DR5 production potentiates the proapoptotic effect of S-TRAIL and thus allows greater therapeutic potential. Accordingly, it is unsurprising that experiments in which S-TRAIL is combined with a proteasomal inhibitor, such as bortezomib, show improved survival in models with bortezomib added on top of TRAIL [80].

One particularly interesting observation in the study by Balyasnikova et al. is that the membrane-bound version (denoted mTRAIL) was more efficacious than the soluble variant, as glioma cells were more susceptible to apoptosis after co-culture with the latter [80]. In discussing their results, the authors hypothesized that this may have been due to the membrane-bound version (mTRAIL) being more difficult to internalize within the target cell than the soluble version (S-TRAIL). Interestingly, Krohlhaas et al. had demonstrated that receptor-mediated endocytosis of TRAIL actually weakens—rather than strengthens—TRAIL’s apoptotic signal; therefore, the increased effectiveness of mTRAIL may be attributable to decreased internalization and thus greater efficacy [83].

Investigators have also trialed a number of additional combinations. For instance, by combining S-TRAIL with temozolomide, a DNA alkylating agent, researchers were able to reduce tumor progression in TRAIL-resistant cell lines [78]. Another combination includes the usage of lipoxygenase inhibitors in addition to TRAIL-secreting mesenchymal stem cells. Lipoxygenase is an enzyme catalyzing the formation of leukotrienes, which are well known contributors to tumor growth [84]. By inhibiting their activity using a lipoxygenase inhibitor (MK886), investigators were able to demonstrate enhanced levels of apoptosis in both TRAIL-resistant and TRAIL-sensitive cell lines [85].

## 6. Enzyme-Prodrug System

An alternative therapeutic approach involving NSCs is to reprogram them such that they express the enzymes required for the activation of specific prodrugs. These specific prodrugs, once they reach the tumor—as a result of NSC migration—will then be activated by these enzymes at the particular location of the tumor, thereby allowing us to selectively release the toxic drug metabolites precisely where they are needed [53,86,87].

One prodrug is ganciclovir, a guanosine analog that inhibits DNA polymerase. This drug, traditionally used as an antiviral, is activated by phosphorylation via the enzyme thymidine kinase. By genetically altering NSCs such that they express thymidine kinase, we can ensure the activation of ganciclovir near the tumor [87]. Another promising prodrug is flucytosine, which requires cytosine deaminase in order to form fluorouracil, the active compound. Fluorouracil can then inhibit DNA polymerase activity. Animal studies have shown that both these prodrugs result in prolonged mice survival in addition to a reduced tumor burden [53,86].

## 7. Viral Vectors

An intriguing novel approach involves the use of SCs as delivery vehicles carrying viruses that can replicate in, and subsequently kill, tumor cells. Injecting replication-competent adenoviruses allows us to make use of this tumor-toxic effect and thus theoretically alleviate the tumor burden. However, one major downside to this approach is that a simple injection of viral particles does not allow a satisfactory degree of tumor penetration. Thus, there is enormous potential in combining stem cells, which can hone in on the target lesion, and toxic adenoviruses that can then kill said targets. Indeed, using mesenchymal stem cells (MSCs), Sonabend et al. were able to deliver a 46-times-higher dose than by simply injecting the viral particle alone [88]. Yong et al. were able to demonstrate significantly improved survival for mice injected with adenovirus-infected mesenchymal stem cells, with the median survival increasing from 42 days in the control group to 75.5 days in the treatment group [89]. Comparing MSCs to NSCs, Ahmed et al.’s results demonstrate the superiority of NSCs [90]. The authors showed that, despite both cells being well-capable of acting as viral vehicles, NSC-treated mice exhibited significantly longer survival than the MSC-treated group (68.5 vs. 44 days, respectively). In addition, MSCs showed greater degrees of migration to negative controls, indicating that NSCs may have a more specific affinity. Based on these results, it is clear that NSCs are, overall, more effective vehicles of oncolytic adenoviridae in gliomas.

Other viral candidates include the herpes simplex virus (HSV), which has been trialed in several studies. Several mutant variants have been described. By mutating certain pathogenic genes in HSV, we can ensure its safety and limit its pathogenicity in humans. One such key gene is Gamma 34.5, which is highly important for the virus’ pathogenic effect. This gene allows HSV to prevent interferons from shutting off protein synthesis. As the ability of interferons to halt protein synthesis is a key mechanism by which our immune system combats viruses, this gene plays a vital role in HSV’s damaging effect. By deleting it, we lessen the risk of adverse effects [91,92]. However, this comes at the cost of limiting HSV’s replication capacity, thereby limiting its therapeutic effect. This type of oncolytic HSV (oHSV) is named HSV1716 [93].

Further developments have led to the development of the G207 type of HSV, which carries an additional mutation in the UL39 gene. This mutation impairs the activity of viral ribonucleotide reductase (RR). RR is highly active in tumor cells; therefore, G207 oHSV, which lacks its own viral RR, can make use of tumor cell RR. As such, this mutation serves to enhance the selectivity of oHSV for tumor cells and, at the same time, attenuate its pathogenicity to the host [94]. More recently, G47 oHSV, which carries an ICP47 deletion, has been trialed. This deletion allows increased MHC1 activity and therefore increases lymphocytic tumor infiltration [95]. 

A different prodrug/enzyme modality is based on the transplantation of allogeneic human NSCs that can express the enzyme carboxylesterase (CE) for glioblastoma management. This method is used along with parenteral treatment with irinotecan (also called CPT-11), which is a prodrug converted by CE to SN-38 (a topoisomerase I inhibitor) [27].

## 8. Other Potential Approaches and Considerations

Loading NSC with mesoporous nanoparticles that contain a chemotherapeutic drug named doxorubicin can be used to treat malignant glial cells’ uncontrolled proliferation [96]. It is thought that the death of NSCs leads to the excretion of these nanoparticles into glioma [96]. Other particles were also used with promising results such as NSC-loaded gold nanorods [97].

Pseudomonas exotoxin (PE) has the potency of arresting the synthesis of cellular proteins via the modulation of the elongation factor-2 (EF-2). This exotoxin can trigger antitumor properties in both hematologic cancers including Hodgkin’s lymphoma, and leukemia, as well as nonhematologic tumors [98,99,100]. Engineered NSC that can secrete PE-cytotoxins can be used as an immunotoxin delivery method to inhibit GBM growth and recurrence [101].

Engineered NSCs can deliver antiangiogenic thrombospondin (TSP-1) in these regions and consequently attenuate angiogenesis and diminish glioma progression, leading to better survival [102]. Furthermore, oligonucleotide therapeutics (ONTs) can be used in the modulation of gene expression to manage brain malignancies [103]. Exosomes secreted by NSCs may influence the transfer of synthetic ONTs, leading to their rapid internalization and retention by NSCs. This delivery technique is being explored in glioma cells [104].

Finally, it is likely that if NSC-based therapy is to gain clinical utility, it may be in combination with, rather than as a replacement of, other modes of therapy currently forming the cornerstone of glioma treatment, such as radiation and chemotherapy; to that end, Tobias et al. have shown a 46% increase in the median survival of mice treated with the combination of NSCs carrying oncolytic adenoviruses and temozolomide/radiation, with the greatest efficacy being reported in cases where the stem cells are injected before, not after, chemoradiotherapy application [105].

## 9. Clinical Trials and Administration of Therapy

There is a paucity of clinical studies investigating the role of stem cells in the management of gliomas. Portnow et al. conducted the first human study in 15 patients with recurrent high-grade gliomas; the authors of the study report no significant difference in progression-free or overall survival [106]. However, it is important to note that the primary aim of the study was to establish the safety of a single stem cell injection and prove the ability of neural stem cells to mediate an appropriate enzyme-prodrug system rather than measure the effect of the treatment on patient outcomes; hence, the findings of this study serve merely as a proof of concept in supporting the idea that genetically modified stem cells, when injected intracerebrally, are effective in distributing the targeted therapy and are relatively safe [106]. A search of the NIH clinical trials database revealed four active/completed phase I clinical studies. The first investigated neural stem cells that have been genetically modified to release *E. coli* deaminase, an example of an enzyme-prodrug system in which the deaminase converts the prodrug flucytosine into 5-fluorouracil—an antimetabolite that induces cell suicide [107]. The second phase I trial is currently active and investigating the same enzyme-prodrug system in combination with Leucovorin [108]. Furthermore, there are two registered trials exploring the response of high-grade gliomas to stem cells loaded with an oncolytic virus; the first [109] was recently completed with no results posted, while the second [110] is still in the recruitment phase. Results of these clinical trials are highly anticipated as they could potentially revolutionize the current approach to personalized therapy. 

The most commonly discussed methods of stem cell delivery include: Intracerebral, or intraventricular administration. The aforementioned clinical studies use intracerebral (IC) administration; however, there is growing evidence that favors the intraventricular route (IVN). Intracerebral injection has the advantage of providing a direct and tumor-specific distribution [111]; however, it is associated with several limitations. According to Gutova et al., intraventricular administration can overcome the four limitations of intracerebral injection that include: (1) Restrictions on the volume that can be injected, (2) decreased NSC viability due to the lack of a supportive environment—compared to IVN delivery where the CSF provides a less hostile environment, (3) scar formation around the intra-tumoral catheter—which subsequently restricts NSC migration, and (4) limitations due to lack of technical skill for intracerebral catheter placement—as compared to Ommaya reservoir placement [112]; findings by Panciani et al. also support the IVN route as being more optimal [113]. Table 1 summarizes active clinical trials evaluating NSC-based therapy in glioma patients.

## 10. Limitations of Therapy

First, one must note that the effects of stem cells, particularly MSCs, have not been completely uniform in the variety of experimental settings in which they have been trialed. These discrepancies were deeply analyzed by Klopp et al. [114], who have illustrated a number of contradictory results in the literature. The reviewers conclude that some of these differences may be due to the heterogeneity of MSCs used, the difference between fetal and adult MSCs (with the latter showing a greater tumor-promoting effect) [114], as well as the timing of MSC injection, as most studies reporting a growth-promoting effect employed simultaneous injection of MSCs and tumor cells, whereas most of those showing a more inhibitory effect had introduced MSCs at a latter phase in tumor activity. These intriguing results suggest that the particular phase of tumor growth, early versus late, may strongly influence the effect of MSCs [114].

In addition, as with any other animal model of disease, key differences exist between animal models and the actual clinically encountered entities. For instance, animal models of glioblastomas are not as well-established as their human counterparts. This time-difference allows the latter to be relatively well-vascularized, thus supplying greater fuel for tumor growth. In addition, greater lapses of time allow tumors to become relatively more heterogenous genetically, an effect that can frustrate clinicians by endowing tumors with treatment resistance and greater degrees of aggressiveness.

Furthermore, it must be noted that stem cell therapy is not entirely free of risk. For instance, the utilization of viral vectors, such as oHSV, carries the theoretical risk of reactivation. This, however, has been alleviated with the development of strains that have greater mutation burdens and more tumor-specificity, as opposed to older strains. For instance, the older Gamma 34.5 mutant carried a potential risk of transforming into the wild-type variety as it was only this particular mutation that rendered it incapable of harming the host. Further developments, which have created greater safety margins by introducing further mutations, have contributed to increasing the safety of these therapies [94,95]. 

## 11. Conclusions

Using NSC technologies in neuro-oncology is opening new insights for better patients’ management. Although the clinical data are still scarce, the migratory and tumor homing features of NSCs can be exploited to provide an important drug delivery source that may help in targeting malignant cells with a reduced toxicity. Yet, the durability of engineered stem cells effects has to be investigated and enhanced to optimize their killing capacities and to avoid the recurrence of the neoplasm. In addition, as many therapy modalities can be provided by NSCs, the best approaches and the optimal combinations that can provide satisfactory clinical results still have to be identified and applied in glioma research. 

## Figures and Tables

**Table 1:** Active clinical trials evaluating NSC-based therapy in glioma patients.

Registration Number	Stem Cell Type	Approach	Phase	Anti-tumor Agent	Route of Administration	Study Status
NCT01172964	NSC	Enzyme-prodrug	Phase I	5-fluorouracil	Intracerebral	Completed
NCT02015819	NSC	Enzyme-prodrug	Phase I	5-fluorouracil	Intracerebral	Active, not recruiting
NCT03072134	NSC	Oncolytic virus	Phase I	Oncolytic Adenovirus	Intracerebral	Active, not recruiting
NCT03896568	NSC	Oncolytic virus	Phase I	Oncolytic Adenovirus Ad5-DNX-2401	Intracerebral	Recruiting

Data were obtained from NIH. U.S. National Library of Medicine (https://www.clinicaltrials.gov/).
